# Benchmarking of pre-training strategies for electronic health record foundation models

**DOI:** 10.1093/jamiaopen/ooaf090

**Published:** 2025-08-13

**Authors:** Samson Mataraso, Shreya D’Souza, David Seong, Eloïse Berson, Camilo Espinosa, Nima Aghaeepour

**Affiliations:** Department of Anesthesiology, Perioperative and Pain Medicine, Stanford University School of Medicine, Stanford, CA 94305, United States; Department of Pediatrics, Stanford University School of Medicine, Stanford, CA 94305, United States; Department of Biomedical Data Science, Stanford University School of Medicine, Stanford, CA 94305, United States; Department of Computer Science, Stanford University, Stanford, CA 94305, United States; Department of Anesthesiology, Perioperative and Pain Medicine, Stanford University School of Medicine, Stanford, CA 94305, United States; Medical Scientist Training Program, Stanford University School of Medicine, Stanford, CA 94305, United States; Department of Anesthesiology, Perioperative and Pain Medicine, Stanford University School of Medicine, Stanford, CA 94305, United States; Department of Biomedical Data Science, Stanford University School of Medicine, Stanford, CA 94305, United States; Department of Pathology, Stanford University School of Medicine, Stanford, CA 94305, United States; Department of Anesthesiology, Perioperative and Pain Medicine, Stanford University School of Medicine, Stanford, CA 94305, United States; Department of Pediatrics, Stanford University School of Medicine, Stanford, CA 94305, United States; Department of Biomedical Data Science, Stanford University School of Medicine, Stanford, CA 94305, United States; Immunology Program, Stanford University School of Medicine, Stanford, CA 94305, United States; Department of Anesthesiology, Perioperative and Pain Medicine, Stanford University School of Medicine, Stanford, CA 94305, United States; Department of Pediatrics, Stanford University School of Medicine, Stanford, CA 94305, United States; Department of Biomedical Data Science, Stanford University School of Medicine, Stanford, CA 94305, United States

**Keywords:** electronic health record, machine learning, foundation models, cardiovascular disease

## Abstract

**Objective:**

Our objective is to compare different pre-training strategies for electronic health record (EHR) foundation models.

**Materials and Methods:**

We evaluated three approaches using a transformer-based architecture: baseline (no pre-training), self-supervised pre-training with masked language modeling, and supervised pre-training. The models were assessed on their ability to predict both major adverse cardiac events and mortality occurring within 12 months. The pre-training cohort was 405 679 patients prescribed antihypertensives and the fine tuning cohort was 5525 patients who received doxorubicin.

**Results:**

Task-specific supervised pre-training achieved superior performance (AUROC 0.70, AUPRC 0.23), outperforming both self-supervised pre-training and the baseline. However, when the model was evaluated on the task of 12-month mortality prediction, the self-supervised model performed best.

**Discussion:**

While supervised pre-training excels when aligned with downstream tasks, self-supervised approaches offer more generalized utility.

**Conclusion:**

Pre-training strategy selection should consider intended applications, data availability, and transferability requirements.

## Introduction

Foundation models have demonstrated remarkable capabilities in natural language processing (NLP) due to increases in compute, training data, and architectural complexity. The successes of foundation models in NLP have led to a widely used foundation model paradigm. In this paradigm, a large model is pre-trained with a self-supervised, masked language modeling (MLM) objective.^1^ This generalist model is then fine-tuned and used for downstream tasks.^2^ This paradigm has been undoubtedly successful in NLP, including specialized natural language domains as well as in genomics, single cell biology, and clinical informatics.^3–6^

The success of this paradigm relies heavily on large amounts of training data (comprising diverse text from the internet, published literature, and other sources). As EHR databases become increasingly accessible for research, a fundamental question emerges: is this self-supervised pre-training paradigm the optimal approach to leverage such databases most effectively for downstream clinical predictive modeling? While previous research has demonstrated that NLP-inspired self-supervised pre-training approaches can indeed improve predictive modeling performance from EHR data,^6–8^ other studies demonstrate that supervised pre-training improves performance.^9^ Furthermore, recent work found that the NLP FM paradigm does not always confer predictive modeling benefit in other domains.^10^ In many cases, supervised baselines exceed FM-based approaches even though the latter use orders of magnitude more training data. This finding indicates that the specific pre-training strategy can significantly impact the effect of pre-training on downstream performance. Clinical informaticians struggle to select optimal pre-training strategies since no studies have directly compared different approaches, making it challenging to interpret existing evidence. Our work explores this question by directly comparing self-supervised pre-training to supervised pre-training on two separate tasks to better understand the best way to utilize a large EHR database to improve downstream predictive modeling performance.

We first investigated this question through a retrospective study focused on a clinically meaningful task: predicting the risk of major adverse cardiovascular events (MACE) for patients treated with doxorubicin. Doxorubicin is a widely used chemotherapeutic agent known for its cardiotoxic side effects.^11^ Studies have demonstrated that the overall occurrence of doxorubicin-induced cardiotoxicity was approximately 9% among cancer patients, with most cases occurring during the first year after completing chemotherapy.^12^ To assess the generalizability of the models to different tasks, we perform a second study and characterize the ability of these pre-trained models to predict mortality. By evaluating multiple pre-training paradigms on these prediction tasks, we aim to determine the impact of different approaches on downstream predictive accuracy, ultimately informing the development of more effective EHR predictive models.

## Methods

### Cohort selection

This study utilized de-identified patient data from Stanford’s EHR system which uses the Observational Medical Outcomes Partnership (OMOP) data schema.^13^^,^^14^ The pre-training cohort consisted of all patients prescribed any antihypertensive medications (*n* = 405 679 patients). The fine-tuning and evaluation cohort consisted of all patients who received doxorubicin (*n* = 5525 patients). All patients who received both antihypertensive medications and doxorubicin were included *only* in the fine-tuning and evaluation cohort. See the Supplementary Methods for additional detail and justification regarding cohort design.

### Modeling approaches

**Figure 1.**
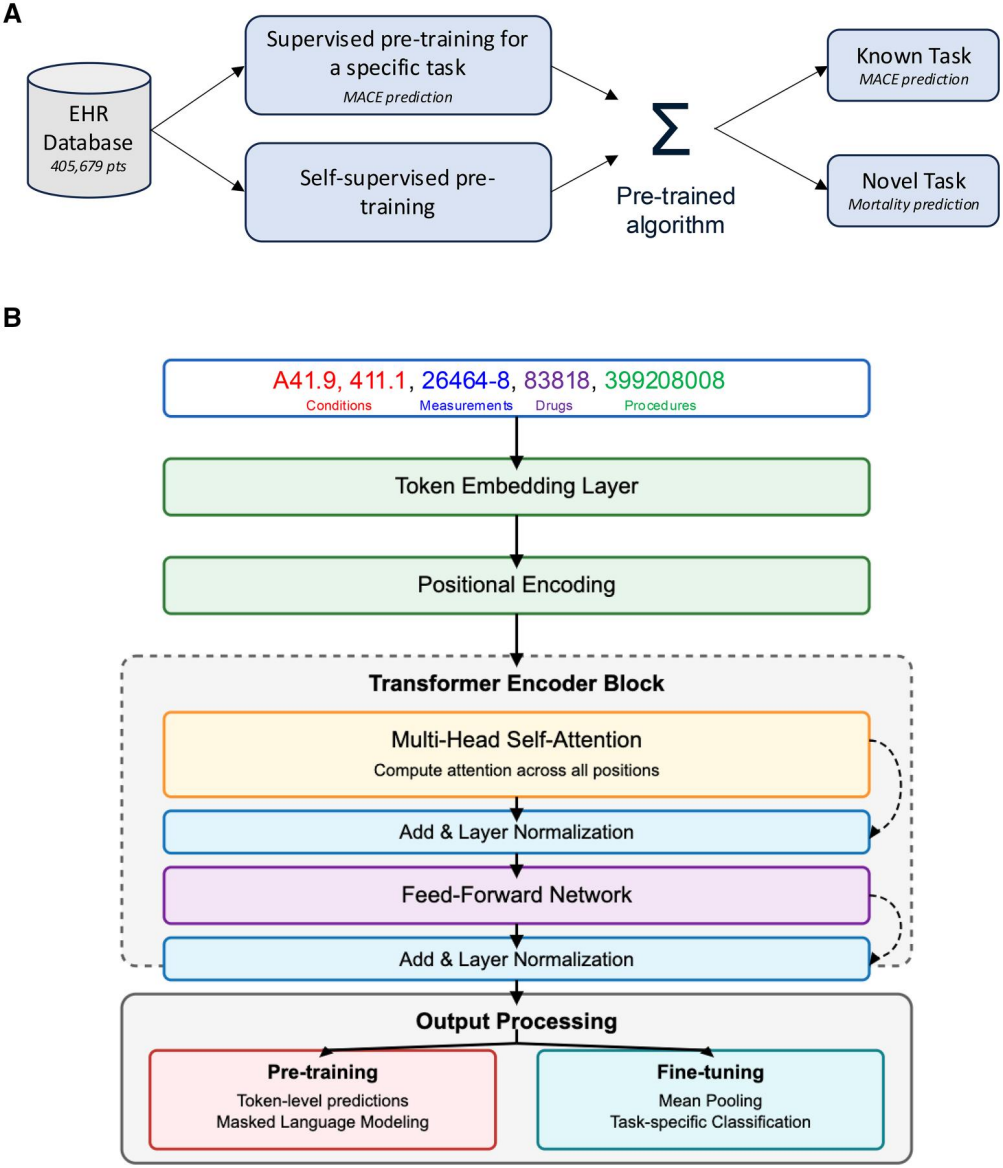
(A) Overall study design. We pre-train models using either a self-supervised approach (masked language modeling) or a supervised approach (standard supervised machine learning to predict 12-month MACE risk). We then apply these pre-trained models to the known task of 12-month MACE prediction or a novel task of 12-month mortality prediction. (B) Data are fed into a transformer based neural network as a sequence of tokens.

The overall goal of the study is to understand how to best utilize a large EHR database to pre-train a model to predict 12-month MACE risk in patients receiving doxorubicin. We subsequently assess the generalizability of these models by measuring their ability to predict 12-month mortality risk. Each of these studies has three experiments: (1) a baseline trained only on the fine-tuning and evaluation cohort, (2) a model pre-trained with a self-supervised objective on the pre-training cohort then fine-tuned on the fine-tuning and evaluation cohort, and (3) a model pre-trained with a supervised objective (MACE prediction) on the pre-training cohort then fine-tuned on the fine-tuning and evaluation cohort (Figure 1A). Each of these three experiments uses the same transformer-based architecture (Figure 1B), described in detail in the Supplementary Methods.

For pre-training, we first performed a grid search across learning rate, dropout, learning rate decay, and model architecture parameters as detailed in **Table S1**. For the self-supervised pre-training, we randomly masked 15% of tokens within each sequence. We used the model to predict each masked token by minimizing the cross-entropy loss. For the supervised pre-training, we trained the model to predict MACE by minimizing the binary cross-entropy (see Supplementary Methods for details). After hyperparameter tuning, we used a single train (70%), test (used for early stopping, 15%), and validation (used to assess performance, 15%) split to pre-train the models.

For fine-tuning and evaluation, we first perform a grid search to optimize learning rate, dropout, and learning rate decay. For the two experiments that utilize the pre-trained models, we also optimize the number of frozen transformer layers (the weights in the other layers are unfrozen to allow them to be fine-tuned). For these experiments, the total number of transformer layers and hidden dimension size are fixed based on optimal hyperparameters from the pre-training stage, as the model architecture must be the same to use the pre-trained weights. For the baseline experiment, we optimize the total number of transformer layers and hidden dimension size as there are no restrictions on these parameters introduced from pre-training as the baseline is trained from scratch within the fine-tuning and evaluation cohort. During fine-tuning, all models minimize a binary cross-entropy objective for either MACE prediction or mortality prediction.

To evaluate performance, we perform 50 iterations with different train-test-validation data splits with the same 70-15-15 split described above. For each patient in the fine-tuning cohort, we computed a final prediction by taking the mean of all predictions made for that patient across the validation sets in which that patient appeared; the models were evaluated based on these final predictions.

## Results

### Model performance

Our evaluation revealed differences in predictive performance across the modeling approaches. The model with supervised pre-training demonstrated superior performance for MACE prediction (Table 1), achieving an AUROC of 0.70 and an AUPRC of 0.23, outperforming all other approaches. The self-supervised pre-training approach showed only minor improvements over the baseline model trained directly on the fine-tuning cohort, suggesting that the self-supervised objective may not have effectively captured the clinical patterns most relevant to MACE prediction. The prevalence of MACE within 12 months of doxorubicin exposure in the fine-tuning and evaluation cohort is 9.4% (*n* = 521 cardiac events) compared to 10.2% (*n* = 41 653 cardiac events) in the pre-training cohort.

**Table 1.** MACE predictive modeling results.

Pre-training	Pre-training objective	AUROC	AUPRC
N	N/A	0.64	0.14
Y	MACE prediction (supervised)	0.70	0.23
Y	Masked token prediction (self-supervised)	0.65	0.15

To evaluate model transferability, we fine-tuned the same pre-trained models for a different clinical task: predicting 12-month all-cause mortality. The prevalence of 12-month mortality is 10.4% (*n* = 574 deaths) in the fine-tuning and evaluation cohort. This analysis is useful to understand if representations learned from pre-training capture only task-specific information, or if the representations are generalizable to other tasks.

For this task, the self-supervised MLM pre-training approach demonstrated the best performance (Table 2). Notably, the supervised pre-trained model—pre-trained to predict MACE—performed worse than the baseline model that was trained only on the fine-tuning cohort without any pre-training. These results highlight an important trade-off: while supervised pre-training delivers superior performance when the pre-training task aligns with the downstream task, it can actually diminish performance when applied to different clinical prediction tasks.

**Table 2.** Mortality predictive modeling results.

Pre-training	Pre-training objective	AUROC	AUPRC
N	N/A	0.79	0.28
Y	MACE prediction (supervised)	0.78	0.26
Y	Masked token prediction (self-supervised)	0.81	0.30

### Interpretability

To understand the performance differences between models, we analyzed feature importance by examining the average attention scores for each token during MACE predictions in the fine-tuning and evaluation cohort. We specifically compared top features after the pre-training phase to top features after the fine-tuning phase for both the supervised and self-supervised approaches.

**Figure 2.**
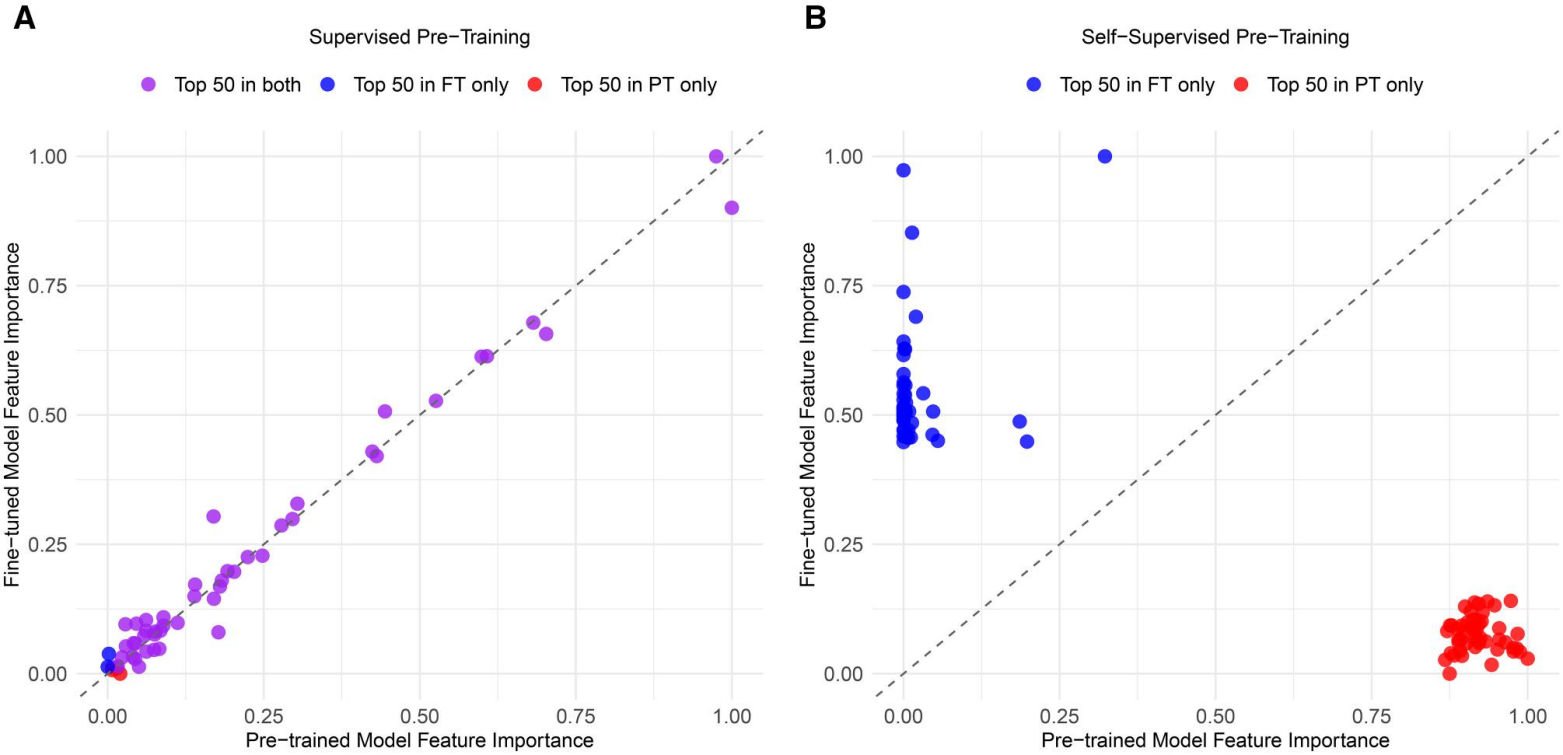
(A) Feature importance for the top 50 features in the pre-trained model (supervised pre-training) before and after fine-tuning, (B) feature importance for the top 50 features in the pre-trained model (self-supervised pre-training) before and after fine-tuning.

This analysis revealed that the feature importance (computed via the attention score for each token when making predictions on patients in the validation set) in the supervised model is fairly consistent before and after fine-tuning (Figure 2A). Forty-eight of the top 50 features in the pre-trained model are still in the top 50 features after fine tuning, with a correlation of 0.997 between the features’ attention scores before and after fine-tuning. We observe a different pattern in the self-supervised model (Figure 2B). The top 50 features before and after fine-tuning are completely different, with no overlap. Additionally, there is a weak correlation of 0.131 between feature importances before and after fine-tuning.

Furthermore, the top features with supervised pre-training were predominantly related to cardiovascular health. After fine-tuning, 4 of the top 5 features in the supervised pre-trained model were cardiovascular-relevant: “cerebral infarction due to embolism of cerebral arteries,” “cardiogenic shock,” “simvastatin 5 MG Oral Tablet,” and “heparin sodium, porcine 1000 UNT/ML Injectable Solution,” with only “attention deficit hyperactivity disorder, combined type” being unrelated. In contrast, the self-supervised pre-training approach demonstrated less alignment with cardiovascular pathophysiology. Its top 5 features after fine-tuning were “sepsis due to Streptococcus pneumoniae,” “heart rate,” “congenital renal cyst,” “congestive heart failure,” and “ERCP study observation”—with only 2 of the top 5 features being directly related to cardiovascular health.

## Discussion

Our evaluation of pre-training strategies for EHR foundation models has yielded several important insights. Most notably, supervised pre-training demonstrated superior performance when the pre-training task matches the fine-tuning and evaluation task. However, self-supervised pre-training provides a benefit over baseline models and has a generalized benefit across a variety of tasks, even though the magnitude of the benefit is not as great as supervised pre-training with task alignment. An important finding is that supervised pre-training on a different task can result in diminished performance. Therefore, a self-supervised approach as used in the NLP paradigm appears to be a good strategy for developing general-purpose foundation models.

As EHR data becomes increasingly accessible, it will be easier to train task-specific models from large datasets. In scenarios in which a clinical informatics team has access to a large dataset for pre-training, task-aligned supervised pre-training has the potential to exceed self-supervised pre-training strategies. We observed this result even though our pre-training population (patients prescribed antihypertensives) differed from the fine-tuning and evaluation population (patients prescribed doxorubicin). However, if the clinical informatics team does not know the downstream task or does not have direct access to the underlying data, self-supervised pre-training would be recommended as it is likely to provide generalized benefit across a wide variety of tasks.

One major limitation of our study is that while MACE and mortality prediction serve as useful medical applications, they remain two specific clinical endpoints; additional studies are needed to evaluate the generalizability of these findings to other clinical prediction tasks. Furthermore, the results may not generalize to endpoints with different prevalence, especially in situations where the prevalence differs between the pre-training cohort and fine-tuning cohort. Another limitation is that our study primarily utilizes a specific architecture; these findings may not generalize to other architectures. The data used in this study come from one medical center and may not generalize to multi-site analyses. Lastly, the performance of the pre-trained models is likely affected by our specific choice of pre-training cohort. It is unknown how a different pre-training cohort may affect these results, and this area warrants further study.

## Conclusion

Our study reveals that supervised pre-training outperforms self-supervised approaches when the pre-training task aligns with the downstream evaluation task. However, self-supervised pre-training still provides benefits over baseline models and offers generalized utility across varied tasks. While supervised pre-training excels when outcome data is available and the evaluation task is known, self-supervised methods remain valuable for the development of general-purpose foundation models that improve performance of clinical risk assessment algorithms. Future research should expand beyond MACE prediction to other clinical endpoints, explore different model architectures, and validate findings across multiple medical centers to establish more generalizable conclusions for EHR foundation model development.

## Supplementary Material

ooaf090_Supplementary_Data

## Data Availability

The data used in this study are electronic health record data and cannot be shared publicly due to Stanford policies.
