# Supplementary material for: Benchmarking of pre-training strategies for electronic health record foundation models
Source: JAMIA Open. 2025 Aug 13;8(4):ooaf090. doi: 10.1093/jamiaopen/ooaf090 (PMC12349770; doi:10.1093/jamiaopen/ooaf090)
Supplement: ooaf090_Supplementary_Data [file ooaf090_supplementary_data.docx]

# Supplementary Methods

## **Cohort Selection**

### **Identification of Antihypertensive Medications**

To identify antihypertensive medications, we included all drugs whose OMOP concept codes were part of the Anatomical Therapeutic Chemical system category for antihypertensives, ACE inhibitors, calcium channel blockers, or lipid modifying agents (OMOP concept codes 21600381, 21601783, 21601744, and 21601853).

### **Cohort Design**

Antihypertensive medications have direct cardiovascular effects and are prescribed to patients with or at risk for cardiovascular disease. By restricting the pre-training cohort to patients prescribed antihypertensive medications, we created a cohort with clinical relevance to our downstream task of MACE prediction, as these patients are more likely to share underlying cardiovascular pathophysiology with patients experiencing doxorubicin-induced cardiotoxicity.

Patients on antihypertensive medications typically have more comprehensive EHR data due to ongoing management of a chronic condition. In the STARR OMOP database, only 0.2% of antihypertensive patients had fewer than 10 clinical events, compared to 22.5% in the general patient population. This restriction ensured our model learned from patients who are likely to utilize Stanford as their primary healthcare provider with significant relevant data.

Additionally, 16.7% of patients in our antihypertensive cohort had cancer diagnoses, providing a representation of patients with similar clinical profiles to our fine-tuning cohort. The prevalence of MACE in our overall patient population was 4.7%, but dropped to 1.3% when excluding patients on antihypertensive medications. By selecting the antihypertensive cohort for pre-training, we obtain a MACE prevalence of 10.2%, which more closely matched the 9.4% prevalence in our doxorubicin fine-tuning cohort.

Our data shows that 7.5% of cancer patients not receiving doxorubicin experienced MACE within 12 months, compared to 9.4% in patients receiving doxorubicin. This increase supports the established relationship between doxorubicin and cardiotoxicity while demonstrating that our pre-training population represents a clinically relevant group for learning cardiovascular risk patterns.

### **Cohort Demographics**

The mean age of patients in the pre-training cohort is 59.6 (standard deviation of 18.1) compared to 53.5 (standard deviation of 17.1) in the fine-tuning cohort. The pre-training cohort is 48.9% female and 51.5% male compared to the fine-tuning cohort which is 63.3% female and 36.7% male. Although the cohorts have different demographic profiles, this is expected due to differences in the cancer patient population compared to the hypertension patient population. For example, doxorubicin is sometimes used to treat breast and ovarian cancers which almost always occur in women so we would expect the fine-tuning cohort to have a greater proportion of women. These differences demonstrate that although the pre-training cohort may differ from the relevant population for our downstream task of predicting MACE due to doxorubicin-induced cardiotoxicity, there is still value in pre-training from this cohort.

## **Modeling Details**

### **MACE Labeling**

The first predictive task was to identify patients at risk of experiencing MACE within 12 months following doxorubicin exposure. Patients were considered positive for MACE if they had an ICD code indicative of acute myocardial infarction, heart failure hospitalization, stroke, or cardiovascular death (any ICD code that starts with 410, 411, 412, 413, 414, 428, 430, 431, 432, 433, 434, 435, 436, 437, I21, I22, I23, I24, I50, I63, I65, or I66) at least 7 days after their first record of doxorubicin administration and less than or equal to 365 days after.

### **Mortality Labeling**

The second predictive task was to predict 12-month mortality risk; patients were considered positive if they had a death record within 12-months following initial doxorubicin exposure. We use the OMOP death table as ground truth for death records.

### **Feature Extraction and Representation**

Patient records were represented as sequences of OMOP concept codes extracted from the measurement, observation, drug exposure, condition occurrence, and procedure occurrence tables. These sequences of codes were directly input into the model. We generated features from data from before the time of initial drug exposure (antihypertensive in the pre-training cohort and doxorubicin in the fine-tuning cohort). Sequences were restricted to the 1024 most recent tokens for computational efficiency; 75.1% of patients fell within this limit.

## **Model Architecture**

### **Overview**

We used a transformer-based architecture to process sequences of clinical events. The architecture consists of a token embedding layer followed by positional encoding. The core of the model includes a transformer encoder with multiple self-attention layers, where each layer comprises a multi-head attention mechanism and a feed-forward network.

### **Embedding and Positional Encoding**

Patient EHR data is processed through a token embedding layer that maps each clinical concept to a dense vector representation where the dimension is a hyperparameter. The embeddings are then combined with positional encodings to incorporate sequence order information, as positional context is critical for interpreting clinical events.

We implemented standard sinusoidal positional encoding as described in Vaswani et al.,^15^ which uses sine and cosine functions of different frequencies:

PE(pos, 2i) = sin(pos/10000^(2i/d_model))

PE(pos, 2i+1) = cos(pos/10000^(2i/d_model))

where pos is the position in the sequence and i is the dimension. This encoding allows the model to understand the relative positioning of clinical events without requiring recurrent connections.

### **Transformer Encoder**

The core of our architecture is a transformer encoder comprising 1-4 layers (number of layers is tuned as a hyperparameter). Each encoder layer contains:

Multi-head Self-attention Mechanism

This component allows the model to attend to different positions in the input sequence simultaneously. For each head, we compute query (Q), key (K), and value (V) projections of the input through separate linear transformations. Attention scores are calculated as:

Attention(Q, K, V) = softmax(QK^T/√d_k)V

where d_k is the dimension of the keys. We use 4 attention heads, each operating on a subspace of dimension d_model/4, allowing the model to jointly attend to information from different representation subspaces.

Feed-forward Network

Following the attention mechanism, each position is processed independently through a two-layer feed-forward network with a ReLU activation:

FFN(x) = max(0, xW₁ + b₁)W₂ + b₂

where the inner layer has dimension 4·d_model.

Residual Connections and Regularization

To facilitate gradient flow and prevent overfitting, we employed residual connections around each sub-layer, followed by dropout (which is optimized as a hyperparameter).

Classification Head

For downstream classification tasks, we aggregate the transformer's output representation across all sequence positions using mean pooling, producing a fixed-length vector. This aggregated representation is then passed through a final linear layer followed by a sigmoid activation function to produce the probability of the target outcome.

### **Model Training**

Self-supervised pre-training uses a masked language modeling objective, where the model is trained to predict tokens based on their context, allowing it to learn useful language representations without labeled data. During pre-training, the model preserves token-level predictions across the full sequence with the output layer dimensioned to predict across the entire vocabulary.

Otherwise during model training we minimized binary cross-entropy loss using the Adam optimizer with tuned learning rates (10^-2 to 10^-4) and weight decay (10^-3 to 10^-4). For pre-trained models, we implemented selective layer freezing, allowing us to fix the weights of early transformer layers while fine-tuning later layers. This approach preserves general representations learned during pre-training while adapting task-specific features during fine-tuning.

Parameter initialization followed standard practices, with embedding weights initialized uniformly within [-0.1, 0.1], and transformer weights initialized using Xavier uniform initialization. To prevent gradient explosion, we applied gradient clipping with a maximum norm of 1.0.

### **Implementation Details and Code**

The code was implemented in Python 3.12.4 with PyTorch 2.5.1. All experiments were run on NVIDIA A40 GPU. The complete code along with a YML file to recreate our computational environment is available at <https://github.com/samson920/EHR_FM_PT>.

## **Supplementary Tables**

Supplementary Table 1. Hyperparameter Grid.

| Hyperparameter | Baseline | Self-supervised Pre-training | Self-supervised Fine Tuning | Supervised Pre-training | Supervised Fine Tuning |
| --- | --- | --- | --- | --- | --- |
| Batch Size | 16 | 32 | 16 | 128 | 16 |
| Learning Rate | {10⁻², 10⁻³, 10⁻⁴} | {10⁻³, 10⁻⁴} | {10⁻², 10⁻³, 10⁻⁴} | {10⁻³, 10⁻⁴} | {10⁻², 10⁻³, 10⁻⁴} |
| Dropout | {0.1, 0.3, 0.5} | {0.1, 0.3, 0.5} | {0.1, 0.3, 0.5} | {0.1, 0.3, 0.5} | {0.1, 0.3, 0.5} |
| Learning Rate Decay | {10⁻⁴, 10⁻³} | {10⁻⁴, 10⁻³} | {10⁻⁴, 10⁻³} | {10⁻⁴, 10⁻³} | {10⁻⁴, 10⁻³} |
| Transformer Layers | {1, 2, 4} | {1, 2, 4} | N/A | {1, 2, 4} | N/A |
| Hidden Dimension | {128, 256} | {128, 256} | N/A | {128, 256} | N/A |
